# A Cross Sectional Survey of Factors Influencing Healthcare Access in Older Women of South India

**DOI:** 10.1093/geroni/igab046.3041

**Published:** 2021-12-17

**Authors:** Sreejith Sudhakar, Justin Jose, Shanuga Cherayi

**Affiliations:** 1 Manipal Academy of Higher Education, Manipal, Karnataka, India; 2 Dream A Dream, Cherupuzha, Kerala, India; 3 Dharmagiri jeevas Social Center, Cherupuzha, Kerala, India

## Abstract

We examined the determinants of healthcare access barriers, treatment-seeking, and self-medication in older women aged 60 years and more, using a cross-sectional survey design. Using a structured interview format, we interviewed 1005 older women from 7 out of 14 districts in the state through a stratified random sampling procedure. Multiple linear regression analysis results reveal that older women's healthcare access barriers significantly increased when they experienced a long duration of multimorbidity alongside poor recognition of autonomy and basic amenities available at health facilities. However, confidentiality, the ability to pay for healthcare expenditure, and the type of health care significantly improved healthcare access. In factors influencing older women's delay in treatment-seeking, optimal instrumental functionality in daily living, optimal quality of life and access to healthcare services significantly reduced delay in treatment initiation. Whereas poor health-seeking behaviors, long duration of multimorbidity, and the quality of basic amenities at hospitals significantly increased treatment initiation delay and explained 13.6% of the variance. In factors influencing older women's use of self-medication, advancing age, living in rural areas, optimal functionality, perception of providers' respect for confidentiality were associated with increased self-medication frequency. Whereas, better wealth status, prompt attention to older women's health needs, and basic amenities at hospitals significantly reduced their self-medication practice. Therefore, the optimal functional abilities, fewer morbidities, and optimal health system responsiveness significantly reduce healthcare access barriers and self-medication while improving older women's treatment-seeking behaviors.

